# New potential biomarker for stratification of patients for pharmacological vitamin C in adjuvant settings of cancer therapy

**DOI:** 10.1016/j.redox.2019.101357

**Published:** 2019-10-23

**Authors:** Rumiana Bakalova, Zhivko Zhelev, Thomas Miller, Ichio Aoki, Tatsuya Higashi

**Affiliations:** a Department of Molecular Imaging and Theranostics, National Institute of Radiological Sciences (NIRS), Institute for Quantum Life Science, National Institutes for Quantum and Radiological Science and Technology (QST), Chiba, Japan; b Group of Quantum-state Controlled MRI, Institute for Quantum Life Science, National Institutes for Quantum and Radiological Science and Technology (QST), Chiba, Japan; c Institute of Biophysics and Biomedical Engineering, Bulgarian Academy of Sciences, Sofia, Bulgaria; d Medical Faculty, Trakia University, Stara Zagora, Bulgaria; e IC-MedTech Co., San Diego, CA, USA

**Keywords:** Cancer, Mitochondria, Ascorbate free radical, NADH:Cytochrome b5 oxidoreductase 3, Redox signaling

## Abstract

Our graphical review expands the analysis of cancer vulnerabilities for high dose vitamin C, based on several facts, illustrating the cytotoxic potential of the ascorbate free radical (AFR) via impairment of mitochondrial respiration and the mechanisms of its elimination in mammals by the membrane-bound NADH:cytochrome b5 oxidoreductase 3 (Cyb5R3). We propose that vitamin C can function in “protective mode” or “destructive mode” affecting cellular homeostasis, depending on the intracellular “steady-state” concentration of AFR and differential expression/activity of Cyb5R3 in cancerous and normal cells. Thus, a specific anti-cancer effect can be achieved at high doses of vitamin C therapy. The review is intended for a wide audience of readers – from students to specialists in the field.

## Highlights

• The ascorbate radical could impair mitochondrial respiration via cytochrome c reduction.
• The ascorbate radical could mediate the imbalance of the coenzyme Q “pool” in cancer cells.
• The selective cytotoxicity of vitamin C in cancer could be mediated by Cyb5R3/VDAC1.
• Low/normal doses of vitamin C act in a “protective mode” for normal/cancer cells.
• High doses of vitamin C act in a “destructive mode” for cancer cells only.

## Graphical abstract

**Figure:**
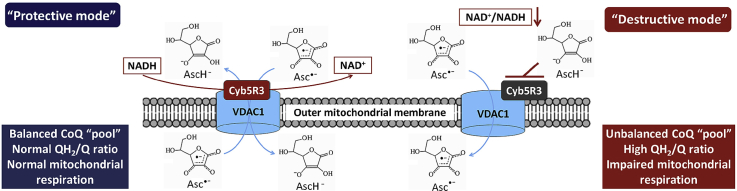

## Abbreviations

AFR: ascorbate free radical
CoQ: coenzyme Q
Cyb5R3: NADH:cytochrome b5 oxidoreductase 3
DHA: dehydroascorbic acid
OMM: outer mitochondrial membrane
VDAC1: voltage dependent anion channel 1

## 1 Introduction

The article of Ngo and colleagues, published in the April 2019 issue of *Nature Reviews Cancer*, raises questions about cancer vulnerabilities for “pharmacological vitamin C” and their significance to the stratification of patients in adjuvant protocols [1]. The authors note that vitamin C as a cancer therapy has a controversial history and much of the controversy arises from the lack of predictive biomarkers for stratification of patients, as well as a clear understanding of the mechanism of action and its multiple targets underlying the anticancer effect [2].

**Fig. 1:**
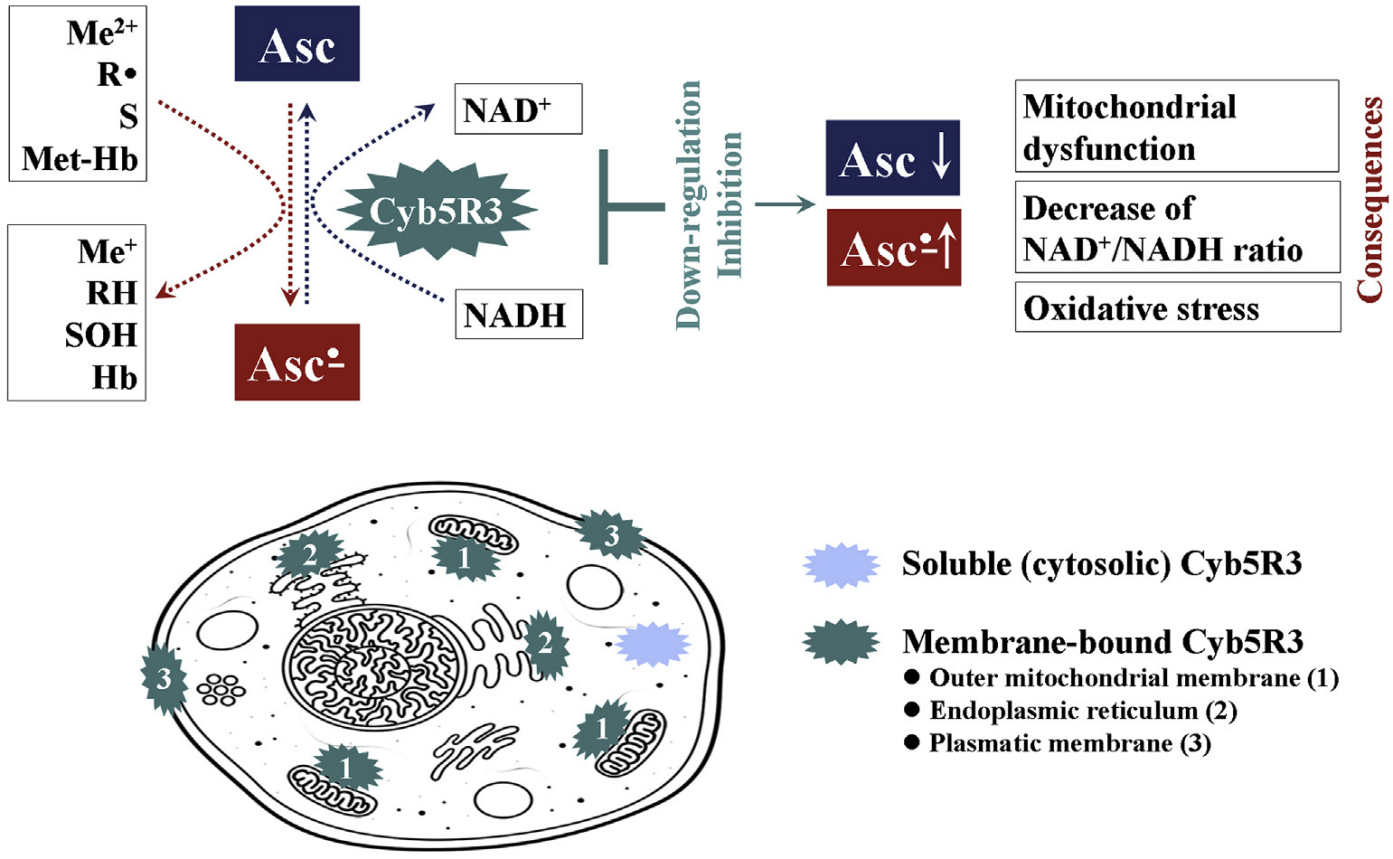
**Mechanisms for production of ascorbate free radical (Asc**^**•**^**) and its elimination in mammals: Main consequences of inhibition of Asc**^**•**^**conversion to ascorbate (Asc).** Asc^•^ is an intermediate product from the oxidation of ascorbate by transition metal ions and/or free radicals. Asc^•^ is a by-product of the conversion of methemoglobin to hemoglobin in erythrocytes, as well as of some enzymatic hydroxylation reactions [27,28]. NADH:cytochrome b5 oxidoreductase 3 (Cyb5R3) catalyzes rapid conversion of Asc^•^ to ascorbate, using cytosolic NADH as an electron donor [4,14]. Thus, Cyb5R3 eliminates Asc^•^, restores the ascorbate “pool” and helps maintain the NAD^+^/NADH ratio in cells. The Cyb5R3 is encoded in two isoforms: (i) soluble, exclusively expressed in erythrocytes, and (ii) membrane-bound, expressed in all other cells and anchored to the outer mitochondrial membrane (OMM), endoplasmic reticulum and plasmatic membrane [4]. The OMM Cyb5R3 enzyme is ubiquitously expressed in all mammalian cells, implying an important role in cell homeostasis. OMM Cyb5R3 uses a cytochrome b5-like hemoprotein of the outer mitochondrial membrane, which is distinct from microsomal cytochrome b5 [13]. Down-regulation and/or inhibition of OMM Cyb5R3 are accompanied by mitochondrial dysfunction, decrease of ATP production and NAD^+^/NADH ratio, and higher sensitivity to oxidative stress [27]. Hb – hemoglobin; Met-Hb – methemoglobin; Me – transition metal; OMM – outer mitochondrial membrane; R^•^ – free radical; RH – non-radical product; S – substrate of enzymatic hydroxylation; SOH – product of enzymatic hydroxylation.

Our article expands the analysis of cancer vulnerabilities for high dose vitamin C in order to define new predictive biomarkers for its therapeutic efficiency. The analysis is based on several indicative facts, that are beyond the focus of the scientists in this field. They are related to the mechanisms for generation and elimination of ascorbate free radical (AFR) in mammals and its mitochondria-mediated cytotoxic potential (Fig. 1).

We assume that the accumulation of high concentrations of AFR in cells during oxidative stress and/or some specific metabolic activity could impair mitochondrial respiration at down-regulation and/or inhibition of NADH:cytochrome b5 oxidoreductase 3 (Cyb5R3), particularly the enzyme located on the outer mitochondrial membrane (OMM Cyb5R3). This can induce cytotoxicity in mammalian cells. Perhaps, this is why enzymes found in plants, that convert ascorbate to AFR (ascorbate oxidase and ascorbate peroxidase), did not evolve in animals and humans [3].

## 2 NADH:semidehydroascorbic acid reductase activity of Cyb5R3

Cyb5R3 belongs to the class of cancer-related and disease-related genes. Cyb5R3 enzyme belongs to the class of potential drug targets and predicted membrane proteins (according to *The Human Protein Atlas*). OMM Cyb5R3 is functionally connected to the voltage dependent anion channel 1 (VDAC1), the most abundant protein of the outer mitochondrial membrane [4]. Both proteins act as one redox-cycling complex that converts AFR to ascorbate. Cyb5R3 is also recognized as a carcinogen detoxification gene [5], although its role in carcinogenesis is not yet well studied. It was found that the OMM Cyb5R3 is overexpressed in cancer cells, protecting them against oxidative stress and induction of apoptosis [[5], [6], [7]]. This enzyme is identified as a key protein in a canonical pathway of the cancerous phenotype associated with mitochondrial dysfunction [7].

VDAC1 is also highly expressed in all cells as a consequence of exposure to various toxic substances and plays a crucial role in the protection against intoxication [4,8]. VDAC1 interacts with both pro-apoptotic and anti-apoptotic factors, which makes it a gate-keeper for mitochondria-mediated cell death or survival signaling pathways [9]. Overexpression of VDAC1 in cancer cells is associated with high metastatic potential, low therapeutic efficiency and poor prognosis [9]. In this case, VDAC1 appears to be involved in protecting mitochondria from oxidative stress, functioning as a pro-survival pathway [8,9].

The key questions are:

● Can pharmacological doses of vitamin C attack cancer cells by inhibiting OMM Cyb5R3/VDAC1 and impairing mitochondrial respiration due to overproduction of AFR?
● Could the expression/activity of OMM Cyb5R3 be a marker for predicting the effectiveness of high dose vitamin C therapy in adjuvant settings?

The crucial role of NADH:semidehydroascorbic acid reductase activity of Cyb5R3 for mitochondrial homeostasis has been demonstrated by several groups [[10], [11], [12]]. Their studies suggest that OMM Cyb5R3 is vital for mitochondrial respiration, resistance to oxidative stress, prevention of cell senescence, and cellular longevity, due to the following characteristics:

● The redox potential of the OMM-bound cytochrome b5 varies from −160 mV to −272 mV, depending on the NAD^+^/NADH ratio [4]. Therefore, OMM Cyb5R3 can catalyze the reduction of a variety of redox-cyclers in addition to AFR, such as: quinone-like compounds, bio-reductive xenobiotics/drug, nitro-compounds, and others. These redox-cyclers can influence the intracellular redox-state.
● Membrane-bound Cyb5R3 helps maintain the cytosolic NAD^+^/NADH ratio, supplying cells with reducing equivalents by transferring electrons from cytosolic NADH to the respective membrane components (electron acceptors and carriers) [4,11,13,14].
● The membrane-bound Cyb5R3 is considered to be one of the major enzymes that maintain the NAD^+^/NADH ratio, by using the coenzyme Q (CoQ) “pools” of the outer mitochondrial membrane and endoplasmic reticulum [4,13,14].

CoQ is most likely the main acceptor of excess electrons, as it is the most abundant membrane-bound redox-active compound in the cell. CoQ is also a key electron carrier in mitochondria. Therefore, outside the inner mitochondrial membrane, CoQ can serve as a “buffer” for excess reducing equivalents, accepting electrons from excess cytosolic NADH via Cyb5R3.

We also assume that ascorbate could serve as a “buffer” of excess reducing equivalents in the intracellular aqueous phase of cancer cells due to their oxidative environment, because ascorbate is one of the most abundant cytosolic redox-active compounds. Steady-state levels of ascorbate are maintained by Cyb5R3 [4,13,14] and they are significantly higher in cancer cells compared to normal cells [1,15].

## 3 NADH:cytochrome c oxidoreductase activity of Cyb5R3

**Fig. 2:**
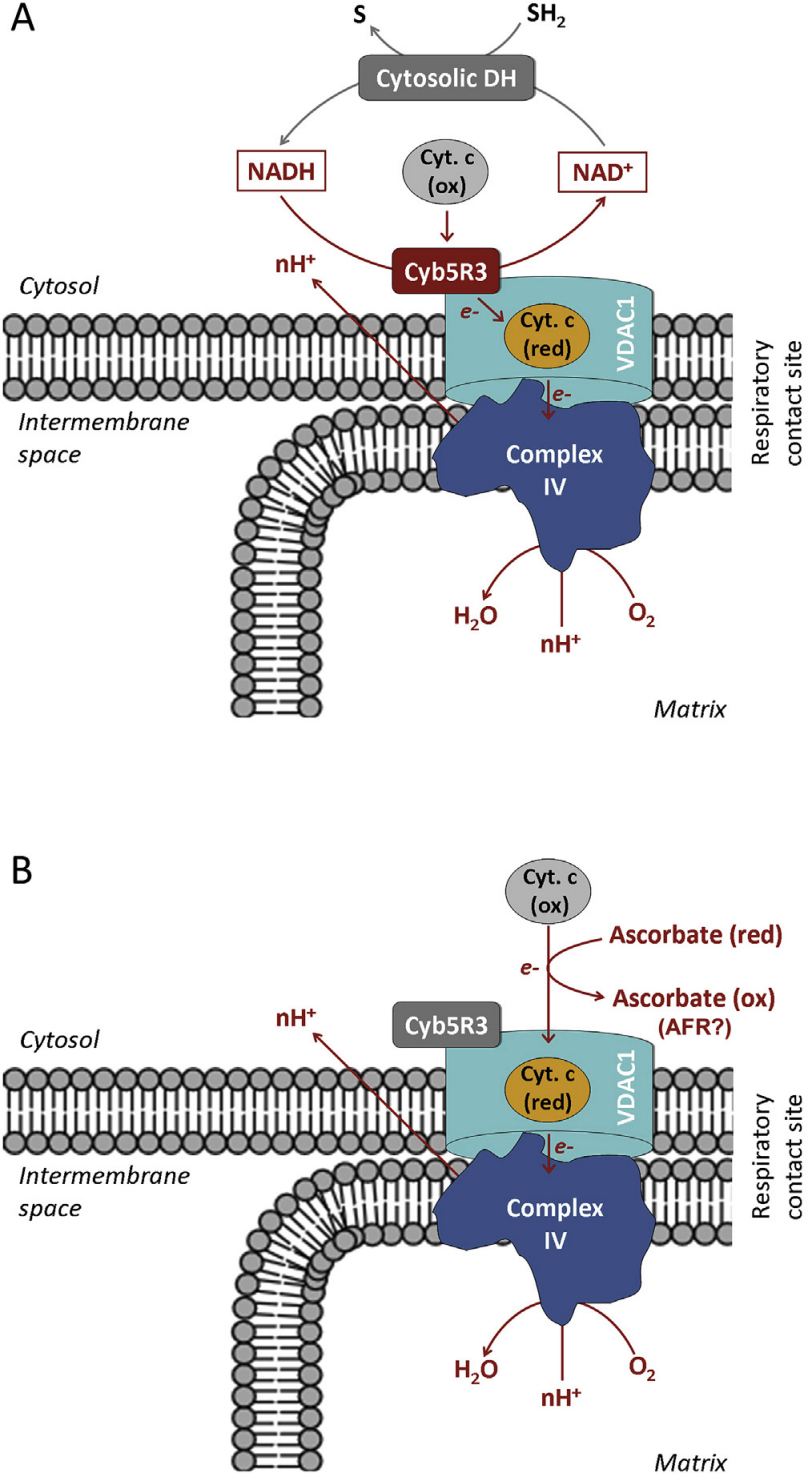
**Schematic representation of the role of small catalytic amounts of external cytochrome c for transferring electrons from cytosol into mitochondria via enzymatic (OMM Cyb5R3-dependent) (A) and non-enzymatic (ascorbate-dependent) pathways (B) [according to data described in La Piana et al.** [[16], [17], [18], [19]]]. The authors have suggested that in physiological conditions, cytochrome c might be transferred in a very limited amount outside the mitochondria so as to promote the activation of Cyb5R3-dependent electron transport pathway. The process is highly dependent on induction of contact sites in the outer and inner mitochondrial membranes (membrane remodeling), as well as on inhibitors of VDAC1. The activity of this Cyb5R3/VDAC1-dependent system becomes functional in removing the excess cytosolic NADH and is essential for cell survival under impairment of the ETC at the level of one of the first respiratory complexes. Since the Cyb5R3-catalyzed electron transfer generates an electrochemical membrane potential associated with the activity of the cytochrome oxidase, it represents an additional pathway for providing energy to cells. This aspect is essential for the cells in which oxidative phosphorylation activity is not properly supported by the respiratory chain. Such phenomenon is observed in cancer cells. (B) The same authors also demonstrate a non-enzymatic induction of this alternative pathway for ATP synthesis in mitochondria). They added ascorbate (instead of NADH) and small catalytic amounts of external cytochrome c to intact mitochondria and detected oxygen uptake, accompanied by cytochrome c reduction and ascorbate oxidation. These processes are dependent on Complex IV and induction of contact sites in mitochondrial membrane, and are sensitive to inhibitors of VDAC1. AFR – ascorbate free radical; DH – NAD-dependent cytosolic dehydrogenases; ETC – electron-transport chain; OMM – outer mitochondrial membrane; S – oxidized substrate; SH_2_ – reduced substrate; VDAC1 – voltage-dependent anion channel 1.

In vitro studies indicate that OMM Cyb5R3/VDAC1 complex possesses mitochondrial NADH:cytochrome c oxidoreductase activity, which is independent of the respiratory chain [[16], [17], [18], [19], [20]]. The authors have reported that OMM Cyb5R3/VDAC1 is responsible for the transfer of electrons from cytosolic NADH into mitochondria, and this process is dependent on Complex IV (Fig. 2A) [[16], [17], [18], [19]]. It is supported by small catalytic amounts of external cytochrome c and accompanied by oxygen uptake, proton pumping and generation of mitochondrial potential. Their studies also demonstrate that cytochrome c-dependent NADH oxidation is strongly inhibited by dextran sulfate (500 kDa) – an inhibitor of VDAC1.

## 4 Ascorbate as a direct reducer of mitochondrial cytochrome c

The same authors have demonstrated that ascorbate can also serve as an electron donor for mitochondria via direct (non-enzymatic) reduction of external cytochrome c, when Cyb5R3 function is impeded (Fig. 2B) [[16], [17], [18], [19]]. This process is accompanied by increased oxygen consumption. Similar results with ascorbate have been reported by Scorano et al. [20] and interpreted as accessibility of ascorbate to cytochrome c present in the intermembrane space, but not to the cytochrome c molecules present in the intracrystal space of mitochondria.

Based on the studies mentioned above, we assume that oxidation of ascorbate by small catalytic amounts of external cytochrome c and/or cytochrome c located in the mitochondrial intermembrane space can lead to AFR production. Ascorbate and AFR exist as anions, which implies their transition between cytosol and mitochondria through the VDAC1. Thus, they can affect mitochondrial respiration at Complexes III-IV – a hypothesis, which is described below in the context of the anticancer effect of high doses of vitamin C.

## 5 “Protective mode” and “destructive mode” of action of vitamin C

Increase of the steady-state concentration of AFR was reported in several conditions characterized by severe inflammation, such as ischemia/reperfusion, carcinogenesis, sepsis, brain injuries, iron overload, and others [[21], [22], [23]].

It has been reported that vitamin C selectively generates AFR in the extracellular fluids in vivo and pharmacologic concentrations preferentially kill cancer cells but not normal cells [15,24]. The AFR concentrations in extracellular fluids measured by EPR are as high as ~250 nM, after parenteral administration of vitamin C in rats in typical human pharmacologic doses [24]. The authors have found that the generation of AFR correlates with ascorbate concentrations in the blood. However, AFR amount in the blood is negligible and can not be detected by EPR. The level of AFR also displays a linear relationship with the production of hydrogen peroxide in the extracellular space in vivo, as well as in isolated normal and cancer cells [15,24]. The authors have assumed that pharmacologic vitamin C is a pro-drug for preferential formation of AFR and hydrogen peroxide in the extracellular space. Thus, high dose vitamin C can selectively suppress proliferation of cancer cells without damaging the normal cells and tissues.

AFR is also detected in living cells and tissues as an intermediate product of ascorbate/dehydroascorbate conversions [25,26]. AFR is an intermediate product from the oxidation of ascorbate by one-electron reduction of transition metal ions and/or interaction with free radicals [1,3]. AFR is a by-product of some vital biochemical reactions. It participates in the recovery of hemoglobin from methemoglobin in erythrocytes, as well as in the enzymatic hydroxylation [e.g., during the synthesis of noradrenalin in the chromaffin granules of dopamine-synthesizing cells, as well as in the synthesis of hypoxia inducible factor 1α, collagen, etc.] (Fig. 1) [27,28]. AFR does not readily react with oxygen or other molecules to generate more reactive radicals and may temporarily accumulate in cells [3]. Keshari et al. have demonstrated that the concentrations of ascorbate, respectively AFR are higher in prostate cancer tissue compared to the respective normal tissue due to overexpression of GLUT3 and SVCT2 proteins, involved in the intracellular delivery of vitamin C [25].

It should be clarified that high intracellular levels of ascorbate, respectively AFR can only be achieved with intravenous vitamin C administration, but not by oral administration [29].

We propose that vitamin C can function in *“protective mode” at low/normal (steady-state) doses* or *“destructive mode” at high doses*, affecting cellular homeostasis, depending on the OMM Cyb5R3 expression/activity and intracellular “steady-state” concentration of AFR. The hypothesis is focused on the effects of AFR in the mitochondrial intermembrane space and the cytosol.

**Fig. 3:**
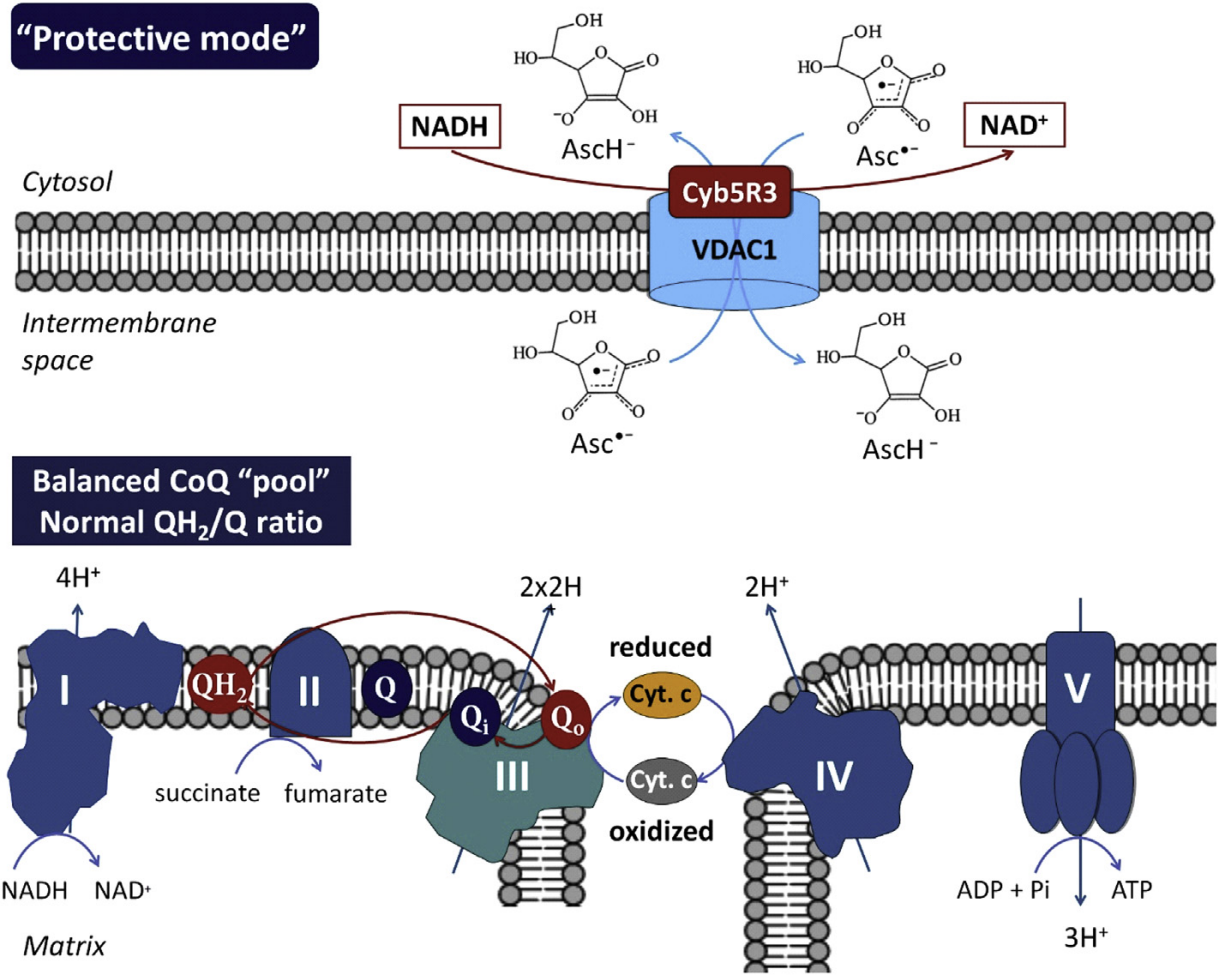
**“Protective mode” of action of the OMM Cyb5R3/VDAC1 at low (steady-state) concentrations of vitamin C in normal cells (at normoxia)**. OMM Cyb5R3/VDAC1 converts ascorbate free radical (AFR; Asc^•^) to ascorbate (AscH^-^), using cytosolic NADH as an electron donor. This prevents effects on mitochondrial respiration and all complexes operate in a “normal mode”, creating a proton gradient and leading to normal ATP synthesis [33]. The CoQ “pool” is balanced and the Qo site of Complex-III transfers one electron to cytochrome c and the second electron to ubiquinone in the Qi site. VDAC1 – voltage dependent anion channel 1; Cyb5R3 – NADH:ferricytochrome b5 oxidoreductase 3; OMM – outer mitochondrial membrane; Cyt. c – cytochrome c.

In normal cells (Fig. 3), low concentrations of AFR should be generated due to the low (steady-state) intracellular levels of vitamin C, as a result of: (i) relatively low expression of vitamin C transport proteins [1,30]; (ii) low steady-state levels of ROS and normal levels of reducing equivalents [31]. Both factors are responsible for the limited production of AFR. Since AFR is relatively stable, it could be rapidly eliminated by the OMM Cyb5R3/VDAC1. This prevents effects on mitochondrial respiration and all complexes will operate in a “normal mode”. Electrons are transferred from Complex-III's Qo site of the CoQ “pool” to cytochrome c, creating a proton gradient and leading to normal ATP synthesis [32]. This model is typical for normal (non-cancerous) cells. It was recently reported that pharmacological (high dose) vitamin C can also protect normal tissues in cancer treatment [33]. The authors have demonstrated that pharmacological vitamin C (intravenous infusion) radiosensitizes pancreatic tumors, but inhibits radiation-induced damage to surrounding normal tissues.

In cancer cells, relatively high “steady-state” levels of vitamin C can be achieved due to overexpression of its transport proteins [1,30]. Cancer cells are also characterized by permanent oxidative stress and redox imbalance, accompanied mainly by overproduction of superoxide, hypoxic environment and overproduction of cytosolic NADH due to up-regulation of the aldehyde dehydrogenase pathway [31,[34], [35], [36], [37]]. These distinct characteristics suggest increased production of intracellular AFR at “steady-state” (non-therapeutic) levels of vitamin C in cancer cells, compared to normal cells. On the other hand, AFR should be rapidly eliminated in the mitochondrial environment of cancer cells due to overexpressed Cyb5R3/VDAC1 [[5], [6], [7],^9^] and high cytosolic levels of NADH [31,34] as an electron donor. Overexpression of the Cyb5R3/VDAC1 appears to be a compensatory mechanism that protects cancer cells and their mitochondria by regulating AFR accumulation at “steady-state” conditions, while also maintaining the cytosolic NAD^+^/NADH ratio.

**Fig. 4:**
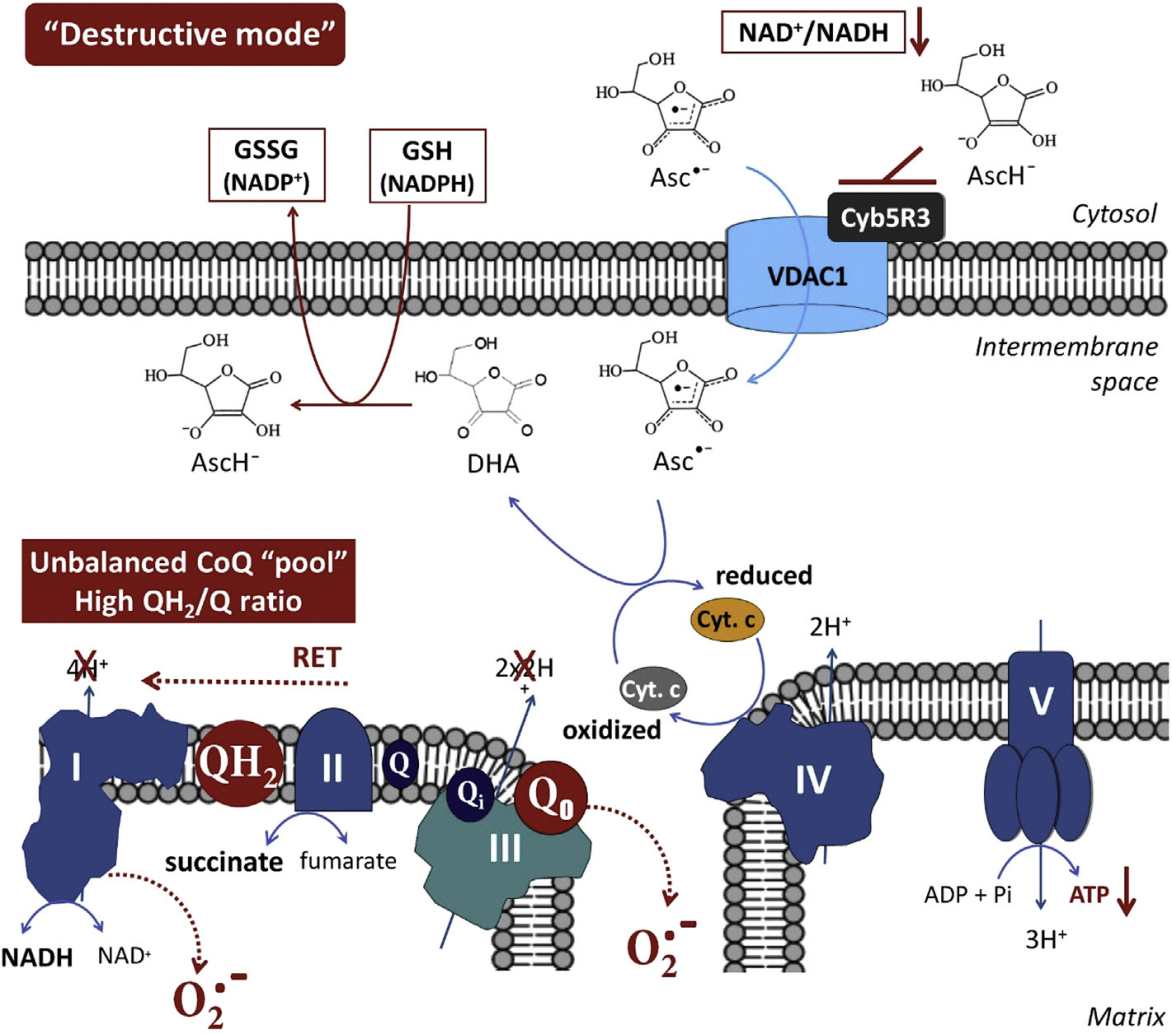
“**Destructive mode” of action of ascorbate free radical via the OMM Cyb5R3/VDAC1 at high (“therapeutic”) concentrations of vitamin C in cancer cells (at normoxia)**. High intracellular concentration of ascorbate may induce Cyb5R3 “end-product inhibition”, accompanied by elevated levels of ascorbate free radical (AFR; Asc^•^) in the mitochondrial intermembrane space and decreasing of NAD^+^/NADH ratio in the cytosol. AFR may transfer one electron to oxidized cytochrome c, causing a partial (or complete) arrest of electron flow between Complex-III and Complex-IV. Rapid reduction of cytochrome c by AFR effectively competes with and perturbs the proton pumping at Complex-III, as well as the temporal electron transport provided by CoQ cycles of Complex-III. Precisely timed redox sequences responsible for the efficient flow of electrons from Complex-III to oxidized cytochrome c are vulnerable to AFR flux. This drives the CoQ “pool” to a more reduced (overcharged) form. This also causes the lifetime of semi-ubiquinone in the Qo pocket to lengthen, allowing oxygen to accept the second electron from ubiquinol during the second step of the CoQ cycle, yielding superoxide. This can interfere with the timing of the Qo/Qi cycle and consequently the CoQ “pool” redox balance, impairing mitochondrial respiration. A highly reduced CoQ “pool” inhibits proton pumping yielding up to 80% lower ATP plus higher levels of superoxide. When Complex-III is blocked, succinate (from the citric acid cycle) builds up, mitochondrial membrane potential rises, and the CoQ “pool” becomes unbalanced. Reverse electron transport (RET) to Complex-I is driven, which causes a superoxide “burst” [59,60]. RET is also accompanied by synthesis of succinate and NADH. We propose that the “destructive mode” caused by high-dose vitamin C may contribute to RET not only during tissue reperfusion at angiogenesis, but also during tumor hypoxia and normoxia in cancer cells. In addition, the reduction of cytochrome c by AFR results in production of DHA. DHA is converted to ascorbate by glutathione, which provokes a depletion of reducing equivalents in cancer cells – a crucial factor for their survival. VDAC1 – voltage dependent anion channel 1; Cyb5R3 – NADH:ferricytochrome b5 oxidoreductase 3; OMM – outer mitochondrial membrane; Cyt. c – cytochrome c; RET – reverse electron transport; DHA – dehydroascorbate; AcsH^−^ – ascorbate in anion form; Asc^•^ – ascorbate free radical.

The first consequence of the high-dose “therapeutic” vitamin C is to selectively elevate its concentration in cancer cells [1,29]. We suppose that high “therapeutic” intracellular concentration of ascorbate in cancer cells is then likely to induce “enzyme (Cyb5R3) end-product inhibition” – a negative feedback, regulating any enzymatic pathway (Fig. 4). The inhibition of OMM Cyb5R3 will result in the accumulation of high amounts of AFR under the permanent oxidative stress within these cells.

In the mitochondrial intermembrane space, AFR may transfer one electron to cytochrome c, causing a partial (or complete) arrest of electron flow between Complex-III and Complex-IV. It has been shown that the rate of reduction of cytochrome c by AFR, with production of DHA, is orders of magnitude faster than the reduction of cytochrome c by ascorbate [38].

Rapid reduction of cytochrome c by AFR effectively competes with the electron transport from the CoQ cycle of Complex-III to the cytochrome c, which perturbs the proton pumping. Competitive inhibition by AFR, by-passing Complex-III to cytochrome c, drives the CoQ “pool” to a more reduced (overcharged) state. If this overcharging phenomenon occurs, there will be insufficient ubiquinone, available for binding at the Qi site. This unbalances the Qo/Qi cycle. It also increases the lifetime of semi-ubiquinone in the Qo pocket, allowing oxygen to accept the second electron from ubiquinol during the second step of the CoQ cycle, yielding superoxide (Fig. 4) [39].

Briefly, we propose that a high level of AFR in the intermembrane space of mitochondria blocks Complex-III, at least partially, via unbalancing the CoQ “pool” and impairing mitochondrial respiration in vitamin C-treated cancer cells. We further propose that the damaging effect of high-dose vitamin C on cancer cells is similar to the mechanism of ischemia-reperfusion injury (IRI) or reoxygenation damage [40]. In IRI, the mitochondrial CoQ “pool” becomes highly reduced by stopped flow through Complex-III.

It is interesting to note that this mechanism can influence the ATP level in different directions. McGuire et al. (2013) have analyzed the ATP in cultured cancer cells (RT4) treated for 1-h with ~8 mM vitamin C, washed and re-suspended in a fresh vitamin C-free medium [41]. The authors have found a slight increase of ATP within 4 h after washing of cells, but ATP decreases on the 5th hour. Uetaki et al. (2015) and Lim et al. (2016) have reported that vitamin C in doses 0.5–15 mM markedly decreases ATP and GTP steady-state levels in cultured cancer cells (AGS, MCF7, HT29) during 2–4 h incubation [42,43]. Buranasudja et al. (2019) have detected a depletion of ATP in high dose vitamin C-treated pancreatic cancer cells, leading to subsequent cell death [44]. The authors explain this finding with the increased energy demand due to hyperactivation of PARP1 and DNA repair, which are ATP-consuming processes. They assume that disruption of bioenergetics is a secondary factor in the vitamin C-mediated cytotoxicity. Gonzalez et al. (2010) also discuss that vitamin C may affect mitochondrial respiration and ATP in different directions depending on the dose [45].

Obviously, ATP steady-state level in vitamin C-treated cells depends on the cell type, vitamin C concentration, duration of treatment, and balance between ATP production and ATP demand. In the case of cancer cells, low doses of vitamin C and/or short-term treatment may induce a slight increase of mitochondrial ATP due to slightly accelerated operation of Complex-IV without significant effect on the other respiratory complexes. On the other hand, high doses of vitamin C in cancer cells may induce a significant reduction of mitochondrial ATP and overproduction of superoxide due to blocking of Complex-III, subsequent induction of reverse electron transport and oxidative disintegration of ETC.

An interesting clinical case shows that in non-cancer cells with mitochondrial deficiency, high dose vitamin C could increase ATP production [46].

It is generally accepted that cancer cells use glycolysis as a source of ATP (Warburg effect) and do not rely on mitochondrial respiration for ATP production. However, recent studies demonstrate that the majority of ATP in cancer cells is produced by mitochondria and some tumors show heavy dependence on oxidative phosphorylation [[47], [48], [49]]. Many solid tumors are poorly perfused and have a limited supply of glucose, but enough oxygen to generate mitochondrial ATP [50]. The ETC is able to function optimally at oxygen levels as low as 0.5% [51]. Therefore, blocking mitochondrial ATP production will induce cell death in poorly perfused tumors.

Even ignoring the dependence of cancer cells on mitochondrial ATP, a functioning of Krebs cycle (at Complex-II) is necessary for the supply of metabolites for synthesis of nucleic acids, fatty acids, etc. [52]. Blocking the ETC will stop this supply and suppress proliferation. Krebs cycle metabolites (such as succinate, fumarate, itaconate …) are also coupled with non-metabolic signaling in cancer and immune cells, which is crucial for cancer progression and invasion [52,53].

The key consideration in targeting mitochondria by high dose vitamin C (via AFR) is that normal cells rely mostly on mitochondrial respiration for ATP production. As it was mentioned above, high concentrations of AFR can not be reached in normal cells, especially in vivo in the absence of oxidative stress, due to lower expression of vitamin C transport proteins [1,15,29] and normal functioning of Cyb5R3 [5,11]. However, normal cells should also be vulnerable to high concentrations of AFR generated under oxidative stress. Perhaps, this is a part of the dual effect of vitamin C, which can act as antioxidant or prooxidant, depending on the environment [54].

**Fig. 5:**
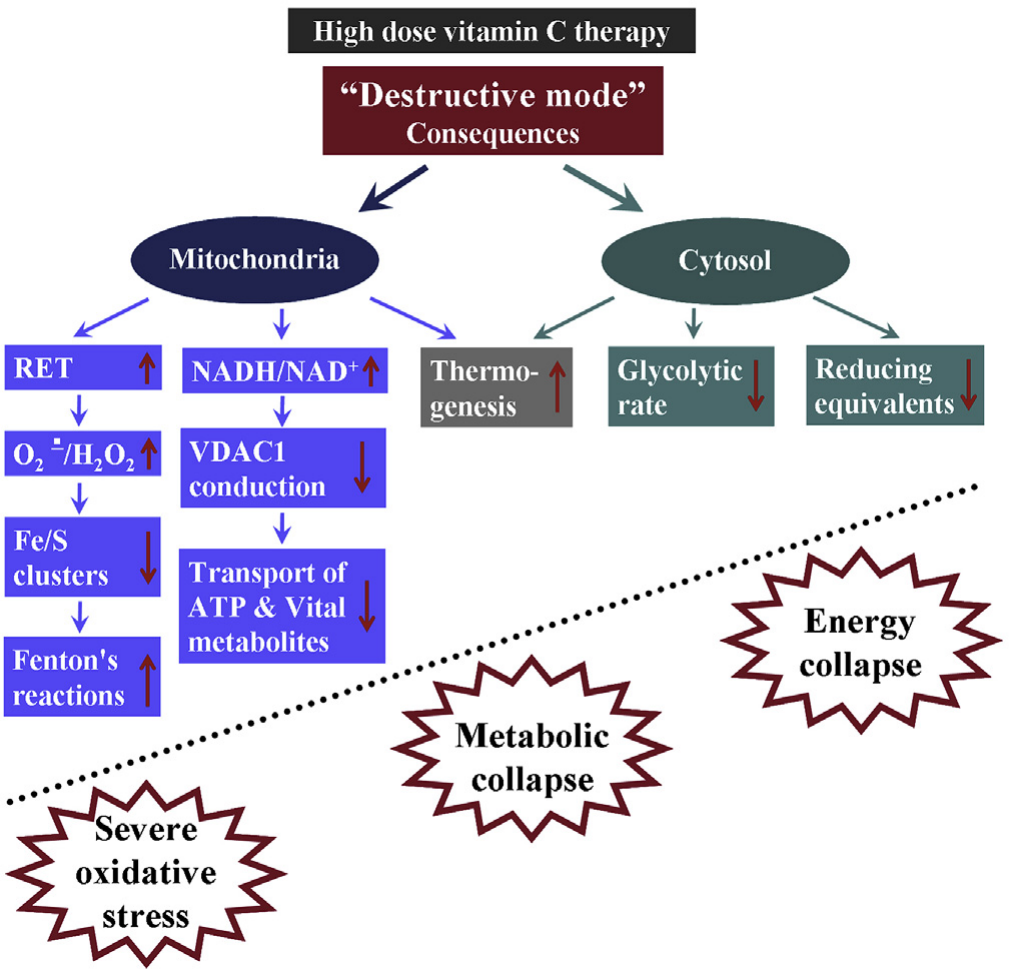
**Consequences of AFR-mediated “destructive mode” of action of high dose vitamin C therapy in cancer**. (a) Induction of reverse electron transport in the respiratory chain and acceleration of superoxide production at Complex-I and Complex-III [34,60]. Degradation of Complex-I when CoQ “pool” is unbalanced, and Complex-III is dysfunctional [60]. In hypoxic conditions (common to many types of cancer cells), these redox reactions occur even faster, exponentially increasing superoxide production [34,60,61]. Superoxide can be converted to hydrogen peroxide, which is considered as a cytotoxic and onco-suppressive reactive oxygen species [15,24,31,35]. (b) Superoxide-mediated destruction of Fe/S clusters from the mitochondrial complexes and induction of Fenton's reactions [62,63], in the presence of AFR and/or ascorbate. (c) Increase of NADH/NAD^+^ ratio when Cyb5R3 is inhibited. NADH reduces conduction of VDAC1 by partial channel block and/or modulation of its activity [64]. As ATP and many other vital substances pass from the mitochondria into the cytosol through VDAC1 [65], partial inhibition of the VDAC1 would also contribute to the impairment of mitochondrial respiration and decrease of cell viability. (d) The conversion of AFR to dehydroascorbate (DHA) and subsequent reduction of DHA to ascorbate by glutathione- and NADPH-dependent pathways will promote a depletion of glutathione and other reducing equivalents in the cancer cells leading to severe oxidative stress [31]. (e) The reduction of cytochrome c by AFR leads to the transfer of electrons from cytoplasmic NADH to Complex-IV, which is accompanied by production of 1 mol ATP per 1 mol NADH, suggesting the likelihood of thermogenesis [66]. Cancer cells often express heat shock proteins and are very sensitive to elevated temperature of their environment [67]. This may also contribute to converting “cold” tumors into “hot” tumors, an important cell death-facilitating factor potentially from combining high dose vitamin C therapy with applications in immunotherapy [68]. (f) Competitive intracellular delivery of vitamin C via glucose transport proteins decreases the glycolytic rate, leading to further decrease of energy supply (by glycolytic ATP) and increased oxidative stress in the cytosol [69]. RET – reverse electron transport; VDAC1 – voltage-dependent anion channel 1.

The consequences of AFR-mediated “destructive mode” of action of high dose vitamin C therapy in cancer are shown in Fig. 5. All mentioned biochemical events lead to energy and metabolic collapse and severe oxidative stress.

## 6 Concluding remarks and outstanding questions

The high concentrations of cytochrome c in mitochondria [54], as well as the possible existence of dedicated pools of CoQ and cytochrome c (as supported by the “plasticity model” of mitochondria) [55,56] could limit the proposed direct effects of AFR on oxidative phosphorylation, described in Fig. 4. In fact, reported concentrations of vitamin C in cells under normal conditions are significantly higher than those of cytochrome c (~10–30 nmol/mg protein versus ~ 0.1–1 nmol/mg protein) [55]. In this case, ascorbate and AFR concentrations in cancer cells after treatment with high doses of vitamin C should be much higher than cytochrome c concentrations [57]. This supports the possibility of direct electron transfer from AFR to cytochrome c at Complex IV. Remodeling of the mitochondrial membrane, and in particular the contact sites of Cyb5R3/VDAC1/Complex IV to optimize performance, can explain the different efficiency and selectivity of high dose vitamin C therapy when combining with different anticancer drugs (depending on their mechanisms). In addition, the above mentioned consequences of AFR-mediated electron transfer in cancerous mitochondria at high dose vitamin C therapy may explain the induction of apoptosis even without strong inhibition of oxidative phosphorylation and ATP production. The mechanism, described in Fig. 4, may not cause strong cancer cell death, but it can damage the mitochondria, stop proliferation and keep cancer cells latent, so they become vulnerable to the immune system of the intact organism.

The analysis of the literature raises several outstanding questions that need experimental verification:

(1) What are the levels of AFR in vitamin C-treated cancer and normal cells and tissues?
(2) Does AFR impair mitochondrial respiration by reducing cytochrome c, causing an arrest of electron flow between Complexes III and IV, a reverse electron transport and an induction of oxidative stress in the absence or down-regulation of Cyb5R3/VDAC1?
(3) Does the vitamin C in low/normal doses work in “protective mode” in normal and cancer cells? If yes, is this related to maintaining CoQ “pool” and cytosolic reducing equivalents balanced?
(4) Does the vitamin C in high doses work in “destructive mode” in cancer cells, but not in normal cells? If yes, is this related to maintaining CoQ “pool” and cytosolic reducing equivalents imbalanced?
(5) What are the levels of mitochondrial superoxide, succinate and ATP in normal and cancer cells of same origin, treated with low, moderate and high doses of vitamin C? Is there a difference in the levels of mitochondrial superoxide, succinate and ATP in cells with different proliferative index, treated with vitamin C?
(6) Does the effect of pharmacological vitamin C relate to the expression and activity of membrane-bound Cyb5R3/VDAC1? Can high doses of vitamin C attack cancer cells only by inhibiting Cyb5R3/VDAC1 and specific destruction of cancer mitochondria?
(7) What is the impact/significance of Cyb5R3/VDAC1 as a predictive biomarker for stratification of cancer-bearing patients for high dose vitamin C therapy?
(8) Does pharmacological vitamin C have a potential as adjuvant cancer therapy in combination with redox-responsive drugs that affect the Cyb5R3?

Our article is intended to provoke the design of new experiments (in vitro and in vivo) that can provide evidence Pro and Con for the assumptions made, to change the “status quo” in the controversial field of “pharmacological vitamin C”. Clarifying the effects of high dose vitamin C and conventional chemotherapeutics on OMM Cyb5R3 activity could be a prerequisite for validation of a new biomarker for predicting the effectiveness of vitamin C in adjuvant protocols and stratification of patients with good/poor prognosis.

If vitamin C can clinically exploit the pathway presented here, we suggest it offers a new, exciting rationale for cancer studies. Perhaps this orthogonal mechanism of action can safely and more effectively compliment current cancer treatments in the adjuvant setting and help to let the Phoenix fly [58].

## Funding

This study was partially supported by the Japanese Society for the Promotion of Science (JSPS) (Grand-in-aid “Kakenhi C″ granted to R.B.), the IC-MedTech Co. (USA) (ICM/QST grant) and the Japanese Agency for Medical Research and Development (AMED) (Project for Cancer Research and Therapeutic Evolution, P-CREATE, no. 16 cm0106202h0001).

## Authors' contributions

RB and ZZ conceived the idea for the manuscript and produced the first draft. TM was involved in the writing of the subsequent drafts. IA and TH were involved in the critical review of the final version. All authors read and approved the final version of the manuscript.

## Declaration of competing interest

No potential conflicts of interest are disclosed.

## References

[1] Ngo B. (2019). Targeting cancer vulnerabilities with high dose vitamin C. Nat. Rev. Cancer.

[2] Unly A., Kirca O., Ozdogan M. (2015). High-dose vitamin C and cancer. J. Oncol. Sci..

[3] Smirnoff N. (2018). Ascorbic acid metabolism and functions: a comparison of plants and mammals. Free Radic. Biol. Med..

[4] Nikiforova A.B., Saris N.-E.L., Kruglov A.G. (2014). External mitochondrial NADH-dependent reductase of redox cyclers: VDAC1 and Cyb5R3?. Free Radic. Biol. Med..

[5] Blanke K.L. (2014). Polymorphisms in the carcinogen detoxification genes Cyb5A and Cyb5R3 and breast cancer risk in African American women. CCC (Cancer Causes Control).

[6] Lund R.R. (2015). NADH-cytochrome b5 reductase 3 promotes colonization and metastasis formation and is a prognostic marker of disease-free and overall survival in estrogen receptor-negative breast cancer. Mol. Cell. Proteom..

[7] Rajcevic U. (2009). iTRAG-based proteomics profiling reveals increased metabolic activity and cellular cross-talk in angiogenic compared with invasive glioblastoma phenotype. Mol. Cell. Proteom..

[8] Camara A.K.S. (2017). Mitochondrial VDAC1: a key gatekeeper as potential therapeutic target. Front. Physiol..

[9] Leanza L. (2014). Mitochondrial ion channel as oncological targets. Oncogene.

[10] Shirabe K. (1995). A novel point mutation in a 3'splice site of the NADH-cytochrome b5 reductase gene results in immunologically undetectable enzyme and impaired NADH-dependent ascorbate regeneration in cultured fibroblasts of a patient with type II hereditary methemoglobinemia. Am. J. Hum. Genet..

[11] Siendones E. (2014). Membrane-bound Cyb5R3 is a common effector of nutritional and oxidative stress response through FOXO3a and Nrf2. Antioxidants Redox Signal..

[12] Carew N.T. (2017). Abstract 20733: cytochrome b5 reductase 3 is essential for cardiomyocyte function. Circulation.

[13] Ito A., Hayashi S., Yoshida T. (1981). Participation of a cytochrome b5-like hemoprotein of outer mitochondrial membrane (OM cytochrome b) in NADH:semidehydroascorbic acid reductase activity of rat liver. Biochem. Biophys. Res. Commun..

[14] De Cabo E. (2010). Cyb5R3: a key player in aerobic metabolism and aging?. Aging.

[15] Chen (2007). Ascorbate in pharmacological concentrations selectively generates ascorbate radical and hydrogen peroxide in extracellular fluid in vivo. Proc. Natl. Acad. Sci. U.S.A..

[16] Marzulli D. (1999). Modulation of cytochrome C-mediated extramitochondrial NADH oxidation by contact site density. Biochem. Biophys. Res. Commun..

[17] La Piana G. (2005). Porin and cytochrome oxidase containing contact sites involved in the oxidation of cytosolic NADH. Arch. Biochem. Biophys..

[18] Bodrova M.E. (1998). Membrane potential generation coupled to oxidation of external NADH in liver mitochondria. FEBS Lett..

[19] La Piana G. (2003). Cytochrome c-induced cytosolic NADH oxidation, mitochondrial permeability transition, and apoptosis. Arch. Biochem. Biophys..

[20] Scorano L. (2002). A distinct pathway remodels mitochondrial cristae and mobilizes cytochrome c during apoptosis. Dev. Cell.

[21] Buettner G.R., Jurkiewicz B.A., Favier (1995). Ascorbate radical: a valuable marker of oxidative stress. Analysis of Free Radicals in Biological Systems.

[22] Drake I.M. (1996). Ascorbic acid may protect against human gastric cancer by scavenging mucosal oxygen radicals. Carcinogenesis.

[23] Spasojevic I. (2011). The role of EPR spectroscopy in studies of the oxidative status of biological systems and the antioxidative properties of various compounds. J. Serb. Chem. Soc..

[24] Chen Q. (2005). Ascorbate in pharmacological concentrations selectively kill cancer cells: action as a pro-drug to deliver hydrogen peroxide to tissues. Proc. Natl. Acad. Sci. U.S.A..

[25] Keshari K.R. (2013). Hyperpolarized [1-13C]dehydroascorbate MR spectroscopy in a murine model of prostate cancer : comparison with 18F-FDG PET. J. Nucl. Med..

[26] Keshari K.R. (2011). Hyperpolarized ^13^C dehydroascorbate as an endogenous redox sensor for in vivo metabolic imaging. Proc. Natl. Acad. Sci. U.S.A..

[27] Siendones E., Ballesteros M., Navas P. (2018). Cellular and molecular mechanisms of recessive hereditary methaemoglobinaemia type II. J. Clin. Med..

[28] Menniti F.S., Knoth J., Diliberto E.J. (1986). Role of ascorbic acid in dopamine β-hydroxylation. J. Biol. Chem..

[29] Padayatty S.J. (2004). Vitamin C pharmacokinetics: implications for oral and intravenous use. Ann. Intern. Med..

[30] Sagun K.C., Carcamo J.M., Golde D.W. (2005). Vitamin C enters mitochondria via facilitative glucose transporter 1 (Glut1) and confers mitochondrial protection against oxidative injury. FASEB J..

[31] Trachootham D., Alexandre J., Huang P. (2009). Targeting cancer cells by ROS-mediated mechanisms: a radical therapeutic approach?. Nat. Rev. Drug Discov..

[32] Sarewicz M., Osyczka A. (2015). Electronic connection between the quinone and cytochrome c redox pools and its role in regulation of mitochondrial electron transport and redox signaling. Physiol. Rev..

[33] Alexander M.S. (2018). Pharmacologic ascorbate reduces radiation-induced normal tissue toxicity and enhances tumor radiosensitization in pancreatic cancer. Cancer Res..

[34] Kim-Muller J.Y. (2016). Aldehyde dehydrogenase 1a3 defines of subset of failing pancreatic β cells in diabetic mice. Nat. Commun..

[35] Pervaiz S., Clement M.V. (2007). Superoxide anion: oncogenic reactive oxygen species?. Int. J. Biochem. Cell Biol..

[36] Zhelev Z. (2019). “Redox imaging” to distinguish cells with different proliferative index: superoxide, hydrogen peroxide, and their ratio as potential biomarkers. Oxid. Med. Cell. Long..

[37] Bakalova R. (2013). Tissue redox activity as a hallmark of carcinogenesis: from early to terminal stages of cancer. Clin. Cancer Res..

[38] Paciolla C., De Gara L. (1991). Reduction of cytochrome c by ascorbic free radical. Boll. Soc. Ital. Biol. Sper..

[39] Bleier L., Drose S. (2013). Superoxide generation by complex III: from mechanistic rationales to functional consequences. Biochim. Biophys. Acta.

[40] Chouchani E.T. (2014). Ischemic accumulation of succinate controls reperfusion injury through mitochondrial ROS. Nature.

[41] McGuire K. (2013). Vitamin C and K3 combination causes enhanced anticancer activity against RT-4 bladder cancer cells. J. Cancer Sci. Ther..

[42] Uetaki M. (2015). Metabolomic alterations in human cancer cells by vitamin C-induced oxidative stress. Sci. Rep..

[43] Lim J.Y. (2016). Vitamin C induces apoptosis in AGS cells via production of ROS of mitochondria. Oncol. Lett..

[44] Buranasudja V. (2019). Pharmacologic ascorbate primes pancreatic cancer cells for death by rewiring cellular energetics and inducing DNA damage. Mol. Cancer Res..

[45] Gonzalez M.J. (2010). Mitochondria, energy and cancer: the relationship with ascorbic acid. J. Orthomol. Med..

[46] Eleff S. (1984). 31P NMR study of improvement in oxidative phosphorylation by vitamins K3 and C in a patient with a defect in electron transport at complex III in skeletal muscle. Proc. Natl. Acad. Sci. U.S.A..

[47] Fan J. (2013). Glutamine-driven oxidative phosphorylation is a major ATP source in transformed mammalian cells in both normoxia and hypoxia. Mol. Syst. Biol..

[48] Varquez F. (2013). PGC1a expression defines a subset of human melanoma tumors with increased mitochondrial capacity and resistance to oxidative stress. Cancer Cell.

[49] Weinberg S.E., Chandel N.S. (2015). Targeting mitochondria metabolism for cancer therapy. Nat. Chem. Biol..

[50] Jain R.K., Munn L.L., Fukumura D. (2002). Dissecting tumor pathophysiology using intravital microscopy. Nat. Rev. Cancer.

[51] Rumsay W.L. (1990). Cellular energetics and the oxygen dependence of respiration in cardiac myocytes isolated from adult rat. J. Biol. Chem..

[52] Tielens A.G.M. (2002). Mitochondria as we don't know them. Trends Biol. Sci..

[53] Ryan D.G. (2019). Coupling Krebs cycle metabolites to signaling in immunity and cancer. Nat. Metab..

[54] Du J., Cullen J.J., Buettner G.R. (2012). Ascorbic acid: chemistry, biology and the treatment of cancer. Biochim. Biophys. Acta.

[55] Lenaz G., Genova M. (2009). Structural and functional organization of the mitochondrial respiratory chain: a dynamic super-assembly. Int. J. Biochem. Cell Biol..

[56] Acin-Perez R., Enriquez J.A. (2014). The function of the respiratory supercomplexes: the plasticity model. Biochim. Biophys. Acta.

[57] Carr A.C., Cook J. (2018). Intravenous vitamin C for cancer therapy – identifying the current gaps in our knowledge. Front. Physiol..

[58] Shenoy N. (2018). Ascorbic acid in cancer treatment: let the Phoenix fly. Cancer Cell.

[59] Dubouchaud H. (2018). Mitochondrial NADH redox potential impacts the reactive oxygen species production of reverse electron transport through complex I. J. Bioenerg. Biomembr..

[60] Robb E.L. (2018). Control of mitochondrial superoxide production by reverse electron transport at complex I. J. Biol. Chem..

[61] Guaras A. (2016). The CoQH_2_/CoQ ratio serves as a sensor of respiratory chain efficiency. Cell Rep..

[62] Clark J.C. (2007). Superoxide-mediated amplification of the oxygen-induced switch from [4Fe-4S] to [2Fe-2S] clusters in the transcriptional regulator FNR. Proc. Natl. Acad. Sci. U.S.A..

[63] Dixon S.J., Stockwell B.R. (2014). The role of iron and reactive oxygen species in cell death. Nat. Chem. Biol..

[64] Zizi M. (1994). NADH regulates the gating of VDAC, the mitochondrial outer membrane channel. J. Biol. Chem..

[65] Okada S.F. (2004). Voltage-dependent anion channel-1 (VDAC-1) contributes to ATP release and cell volume regulation in murine cells. J. Gen. Physiol..

[66] Silva J.E. (2006). Thermogenetic mechanisms and their hormonal regulation. Physiol. Rev..

[67] Calderwood S.K., Gong J. (2016). Heat shock proteins promote cancer: it's a protection racket. Trends Biochem. Sci..

[68] Haasen J. (2017). Converting cold into hot tumors by combining immunotherapies. Cell.

[69] Ma E. (2017). Pharmacological ascorbate induces neuroblastoma cell death by hydrogen peroxide mediated DNA damage and reduction in cancer cell glycolysis. Free Radic. Biol. Med..

